# Genome-Wide Profiling of Enterotoxigenic *Staphylococcus aureus* Strains Used for the Production of Naturally Contaminated Cheeses

**DOI:** 10.3390/genes11010033

**Published:** 2019-12-27

**Authors:** Guerrino Macori, Alberto Bellio, Daniela Manila Bianchi, Francesco Chiesa, Silvia Gallina, Angelo Romano, Fabio Zuccon, Raúl Cabrera-Rubio, Alexandra Cauquil, Déborah Merda, Fréderic Auvray, Lucia Decastelli

**Affiliations:** 1 National Reference Laboratory for Coagulase-Positive Staphylococci including *Staphylococcus aureus*, Istituto Zooprofilattico Sperimentale del Piemonte, Liguria e Valle d’Aosta, Via Bologna 148, 10154 Torino, Italy; alberto.bellio@gmail.com (A.B.); manila.bianchi@izsto.it (D.M.B.); silvia.gallina@izsto.it (S.G.); angelo.romano@izsto.it (A.R.); fabio.zuccon@izsto.it (F.Z.); lucia.decastelli@izsto.it (L.D.); 2 Dipartimento di Scienze Veterinarie, Università di Torino, 10095 Grugliasco, Italy; francesco.chiesa@unito.it; 3 Teagasc Food Research Centre, Moorepark, Fermoy, P61 C996, Ireland-APC Microbiome Ireland, University College Cork, T12YT20 Cork, Ireland; Raul.CabreraRubio@teagasc.ie; 4 European Laboratory for Coagulase-Positive Staphylococci including *Staphylococcus aureus*, Laboratory for Food Safety, ANSES, Université Paris-Est, F-94700 Maisons-Alfort, France; Alexandra.Cauquil@anses.fr (A.C.); Deborah.merda@anses.fr (D.M.); f.auvray@envt.fr (F.A.)

**Keywords:** staphylococcal enterotoxins, annotation, *Staphylococcus aureus*

## Abstract

*Staphylococcus aureus* is a major human pathogen and an important cause of livestock infections. More than 20 staphylococcal enterotoxins with emetic activity can be produced by specific strains responsible for staphylococcal food poisoning, one of the most common food-borne diseases. Whole genome sequencing provides a comprehensive view of the genome structure and gene content that have largely been applied in outbreak investigations and genomic comparisons. In this study, six enterotoxigenic *S. aureus* strains were characterised using a combination of molecular, phenotypical and computational methods. The genomes were analysed for the presence of virulence factors (VFs), where we identified 110 genes and classified them into five categories: adherence (*n* = 31), exoenzymes (*n* = 28), genes involved in host immune system evasion (*n* = 7); iron uptake regulatory system (*n* = 8); secretion machinery factors and toxins’ genes (*n* = 36), and 39 genes coding for transcriptional regulators related to staphylococcal VFs. Each group of VFs revealed correlations among the six enterotoxigenic strains, and further analysis revealed their accessory genomic content, including mobile genetic elements. The plasmids pLUH02 and pSK67 were detected in the strain ProNaCC1 and ProNaCC7, respectively, carrying out the genes *sed*, *ser*, and *selj*. The genes carried out by prophages were detected in the strain ProNaCC2 (*see*), ProNaCC4, and ProNaCC7 (both positive for *sea*). The strain ProNaCC5 resulted positive for the genes *seg*, *sei*, *sem*, *sen*, *seo* grouped in an exotoxin gene cluster, and the strain ProNaCC6 resulted positive for *seh*, a transposon-associated gene. The six strains were used for the production of naturally contaminated cheeses which were tested with the European Screening Method for staphylococcal enterotoxins. The results obtained from the analysis of toxins produced in cheese, combined with the genomic features represent a portrait of the strains that can be used for the production of staphylococcal enterotoxin-positive cheese as reference material.

## 1. Introduction

The advances in sequencing technologies, including next generation sequencing (NGS) have contributed to the revolution of food safety, facilitating the detection and characterisation of foodborne pathogens [1,2,3]. Since their first applications in the phylogenetic analysis of pathogens and spoilage microorganisms, it has been possible to reveal genomic clusters [4] and genes that are crucial for host colonisation and bacterial propagation during infection [5]. Some noticeable examples are the use of genotyping assays for molecular differentiation [6] and the use of new approaches and models for the study of complex microbial communities [7,8]. High throughput DNA sequencing technologies, moreover, can be applied for the study of accessory elements that confer genetic diversity in prokaryotic organisms [9] as well as comparative genomics studies. These approaches have crucial roles enlightening important features, such as antimicrobial resistance [10,11], and for discovering potential virulence factors (VFs) [12,13].

Whole genome sequencing (WGS) provides a comprehensive view of the genome structure and gene content of microorganisms [14,15,16], and is now gradually replacing well-recognised, standardised protocols like PulseNet for its higher sensitivity, specificity, and inter-laboratory comparability, as well as timely resolution when compared with traditional molecular methods [17,18]. WGS has been applied for genomic comparison of microorganisms associated with foodborne outbreaks and for surveillance of high-burden diseases for almost a decade [15], allowing the identification of pathogens in the foods responsible for the infections [15,19,20,21]. In fact, precise gene identifications and detailed virulence portraits can be determined in a single session of sequencing, while bioinformatics analysis for foodborne pathogens provides valuable resources for databases and collections of genomic features [22].

One of the most common foodborne related diseases is the staphylococcal food poisoning (SFP), resulting from the consumption of foods containing sufficient amounts of staphylococcal enterotoxins (SEs) [23]. SFP is associated with enterotoxigenic *S. aureus* (*S. aureus*) strains that may produce one or more heat-stable enterotoxins in food, causing vomiting and abdominal pain in humans [24]. In 2015, 9.9% of all European outbreaks were caused by SEs [25], indicating the importance of surveillance plans and publication of updated reports for risk assessment evaluations. Indeed, the detection of SEs for milk products is a microbiological criterion included into the Commission Regulation (EC) No 2073/2005 [26].

To date, 26 different staphylococcal superantigens (SAgs) have been identified, including the staphylococcal enterotoxins (SEA to SEE; SEG to SEI; SEK; SEM to SET) reported as responsible agents for foodborne outbreaks [27,28,29]; the staphylococcal enterotoxin-like toxins (SElJ; SElL; SElU to SElZ) which are a group of SEs that are not emetic in a primate model, or, their relationship with food poisoning, have yet to be experimentally demonstrated [30,31,32,33]; and the toxic shock syndrome toxin (TSST-1) [34]. Several studies were focused on the genes and the molecular bases of the infections, including emetic studies of newly discovered enterotoxins [35,36,37,38], other virulence factors [39,40] and superantigenic properties of SEls [41]. The genes encoding for *S. aureus* VFS are carried by mobile genetic elements (MGEs) and consist of prophages, plasmids, transposons, and *S. aureus* pathogenicity islands (SaPIs) [42,43], which encode, among the others, enterotoxins and adhesins [23,44]. MGEs mediate their own transfer and integration into new genomic sites and, with a phenomenon called horizontal gene transfer (HGT), also among other bacteria, cause adaptive consequences, such as the transfer and acquisition of antibiotic resistance genes [45]. In this study, six enterotoxigenic *S. aureus* strains were genome-sequenced. A report of their virulence portraits is presented, and the databases used for the detection of the genomic features are described. The strains were also used for the production of naturally contaminated cheeses, suggesting new opportunities for the production of reference materials for inter-laboratory tests, required by regulations [46]. The results obtained from the analysis of toxins produced in cheese, and the comparison with the genomic data, highlighted the requirement of validated methods for the detection of enterotoxins that are more effective. In time, the genes portrait will be translated to provide an effective tool for the detection of the factors responsible for the production of SEs in vivo and predict the enterotoxins that can be produced in complex matrices, which is valuable for surveillance management and corrective action strategies in processing facilities.

## 2. Materials and Methods

### 2.1. Bacterial Study Isolates and Sample Preparation

The experiments were performed using a number of archived strains of *S. aureus* which were maintained in cryogenic vials stock culture beads at −80 °C in two collections: at the European Reference Laboratory for Coagulase Positive Staphylococci, (EURL CPS, Maisons-Alfort, France) and at the Italian Reference Laboratory for CPS (ITRL CPS, Turin, Italy). Six *S. aureus* strains were chosen for this study, representing enterotoxin producers isolated from cheese (five strains) and one strain isolated from a composed salad. Among the six strains, three were isolated in cheeses, which resulted as being responsible for foodborne poisoning involving patients (Table 1).

To regenerate the bacteria, one bead was placed in 10 mL of tryptone soya broth (TSB) (Basingstoke, Oxoid, UK) and incubated statically for 24 h at 37 °C. A total of 10 µL were plated on tryptone soya agar (TSA) (Basingstoke, Oxoid, UK) and incubated for 24 h at 37 °C for checking purity and obtaining single colonies. Species confirmation was performed by a VITEK system GP card (bioMérieux, Marcy l’Etoile, France). Single colonies were re-suspended in TSB and cultured for 18 h at 37 °C and 1.8 mL of the pure cultures were used for DNA isolation with DNeasy UltraClean Microbial Kit (Qiagen, Milan, Italy) following the manufacturer’s protocol. Quality and DNA concentrations were determined with a Qubit 2.0 fluorometer (Invitrogen, Monza, Italy) and Agilent 2100 bioanalyzer (Agilent, Leini, Italy).

### 2.2. Detection of Staphylococcal Enterotoxin Genes by Multiplex PCR Assay, Typing and Antimicrobial Susceptibility Testing

The strains were analysed for the presence of 11 enterotoxins using 2 multiplex PCR assays (mPCR) according to the EURL CPS methods [49,50]. The protocols included the detection of the genes from *sea* to *see* and *ser* for the first mPCR (annealing temperature was 55 °C) and from *seg* to *selj* and *ser* for the second mPCR (annealing temperature was 52 °C); the primers used are reported in Table 2. Five reference *S. aureus* strains were used as positive controls for the SEs genes: *S. aureus* FRIS6 (positive for *sea*, *seb*), *S. aureus* FRI137 (positive for *seg*, *seh*, *sei*), *S. aureus* FRI326 (positive for *see*), *S. aureus* FRI361 (positive for *sec*, *sed*, *ser*), and *S. aureus* HMPL280 (positive for *seg*, *sei*, *selj*, *sep*).

Multilocus sequence typing (MLST) was performed on *S. aureus* isolates targeting seven housekeeping genes (*aroE*, *pta*, *glpF*, *arcC*, *gmk*, *tpi*, and *yqiL*) according to Enright et al. [51]. Strains were further characterised by *spa* typing following the method developed by Harmsen and colleagues [52].

To determine the susceptibility to antimicrobials, Vitek 2 (bioMérieux, Marcy l’Etoile, France) testing was performed using software version 5.04 and the AST-GP79 cards for *S. aureus*, according to the manufacturer’s instructions. The six strains were analysed for the presence of genes *mecA* and *mecC* (methicillin resistant *S. aureus* factors—MRSA) along with the genes *pvl* (Panton-Valentine Leukocidin—PVL) and *spa*, according to the method by Stegger et al. [53].

### 2.3. Whole-Genome Sequencing, and Bioinformatics Analysis

Genomic libraries were prepared using the Illumina Nextera library preparation kit for each strain following the manufacturer’s protocol. WGS was carried out on the Illumina MiSeq platform. The quality of the reads was assessed using FastQC (version 0.11.5) [53]. Specifically, reads were filtered for length, while those with a quality score less than Q30 were discarded using Trimmomatic (version 0.36) [54]. *De-novo* contigs were generated using SPAdes (v.3.9.1) [55], and the quality of each assembled genome was assessed with QUAST (v.4.3) [56]. The assemblies were annotated using Prokka (v.1.11) [57] and RAST [58] for the prediction of coding sequences (CDSs). 

### 2.4. Identification of Virulence Factors and Genomic Analysis

The genomes were interrogated for a pool of 1300 genes, including VFs reported for staphylococci and determined using a combination of a database built for this study (Supplementary Material Database S1) and the PATRIC tool (http://patricbrc.org) (v3.5.41) [59]. The VFs were classified into six functional categories: genes involved in adherence, exoenzymes, host immune evasion factors, iron uptake and metabolism, toxins and transcriptional regulatory elements. Antibiotic resistance genes were detected from the assemblies using ABRicate v0.7 (https://github.com/tseemann/abricate) interrogating the comprehensive antibiotic resistance database (CARD) (https://card.mcmaster.ca). ABRicate was used for the creation of three novel databases built for the detection of SaPIs (Supplementary Material Database S5), plasmids (S7), and genes coding for enterotoxins (S3). The sequences were searched within the genomes of all isolates, consequently, their presence was validated using BLAST+ (v.2.5.0) applying a cut-off value of ≥80% base identity and ≥95% coverage. In silico, MLST was performed using the dedicated server [60] to determine whether any isolates might have been misassigned in the MLST scheme. Bagel3 was used for the detection of genes encoding bacteriocins [61] and prophages sequences were searched to verify the contribution of integrative elements in the structure of bacterial genomes and virulence factors; their sequences were predicted using the PHAge Search Tool (PHASTER) [62]. The annotated genomes were used as input for calculating the pangenome with Roary v.3.12.0 [63] and Anvi’o 2.0.2 [64] and visualized using Phandango [65]. In order to avoid ambiguities in taxonomic inferences, the average nucleotide identity (ANI) comparison of the six genomes was carried out using two independent ANI determination methods: ANI Calculator (http://enve-omics.ce.gatech.edu/ani/index) and EzGenome (http://www.ezbiocloud.net/ezgenome/ani). The genomes of 29 enterotoxigenic *S. aureus* strains were used for the genomic comparison of the six strains presented in this study and included in the constructing of the pan-genome, which was annotated with the use of the Clusters of Orthologous Groups (COGs) database [66]. The genomes were obtained from the assembly database (https://www.ncbi.nlm.nih.gov/assembly/), and the details of the strains are reported in the Supplemental Table S2. All the tools and software were used with default settings if not specified, and data in figures or intext are presented as mean +/− one standard error of the mean unless otherwise indicated. Heatmaps and standard statistical analyses were conducted in RStudio v.1.2.1335 *(R Core Team 2018*). For the construction of the approximate maximum likelihood phylogenetic tree, FastTree was used [67]. In addition, the tool was used for generating the phylogenetic tree and the pangenome matrix.

### 2.5. Production of the Natural Contaminated Cheese and Detection of Preformed Staphylococcal Enterotoxins in Milk and Cheese 

The strains used in this study were tested for the production of SEs in broth culture. Briefly, two colonies from TSA were transferred to 10 mL of TSB and statically incubated at 37 °C for 18 h. The cultures in broths were centrifuged for 5 min at 3500× *g* at 10 °C, and the supernatant and cell debris were removed by filtrationwith 0.22 µm filters. SEs detection was performed with the use of commercially available polyvalent enzyme-linked immunoassay (ELISA) RIDASCREEN SET Total (R-Biopharm AG, Darmstadt, Germany) and enzyme-linked fluorescent immunoassay (ELFA), Vidas SET2 (bioMérieux, Marcy l’Etoile, France). These reference methods can simultaneously detect SEA to SEE in food matrices without differentiating the five SEs [68].

The six strains selected for this study were used for inoculating bovine pasteurised whole milk (GS, Milan, Italy) followed by the production of 12 batches of cheese (two for each strain). For obtaining a batch of SE-positive cheese, each TSA pure culture of the strains were re-suspended in 30 mL of sterile 0.85% NaCl solution obtaining a 3 McFarland unit suspension that was inoculated into 10 L of milk, corresponding to 9 × 10^8^ CFU/mL. The milk was incubated for 18 h at 37 °C, allowing the strain to produce the enterotoxins. After incubation, CPS were enumerated according to ISO 6888-2:1999 [69]. The milk was also tested for the presence of SEs through an extraction step with dialysis concentration followed by qualitative detection using the two validated kits previously described. The liquid rennet (Caglificio Clerici, Cadorago Como, Italy) was added at 1 mg/10 mL concentration, according to the producers’ instructions. The milk was maintained at 37 °C for 1 h for the production of the curd that was eventually broken and transferred into a cheese mould and ripened for 8 days at 10 °C. At the end of the ripening, one portion of rind and one portion of core were tested for CPS and SEs. Rind and core extracted portions were also quantified for SE type A to E following the confirmatory method of the EURL for CPS [68].

## 3. Results

### 3.1. SEs Genes and Molecular Typing

Four of the strains resulted positive for one staphylococcal enterotoxin gene by mPCR: *S. aureus* ProNaCC1, ProNaCC2, ProNaCC4 and ProNaCC6 resulted positive for *sed*, *see*, *sea*, and *seh*, respectively. *S. aureus* strain ProNaCC5 resulted positive for *seg* and *sei,* and ProNaCC7 was positive for the genes *sea*, *sed*, *selj*, and *ser* (Table 3). The *spa* types and MLST resulted unique for each strain, except for the strains *S. aureus* ProNaCC1 and ProNaCC7, which belonged both to sequence type 8 (ST-8) but different *spa*-type (t2953 and t3802, respectively). All the strains resulted negative for the genes *mecA*, *mecC* and *pvl*, and were, therefore, predicted as methicillin-sensitive *S. aureus* (MSSA). 

In addition, the strain ProNaCC4 resulted a new *spa* type therefore, both the forward and reverse sequences of the gene *spa* were submitted to the server https://spa.ridom.de/index.shtml and a unique repeat succession was detected, assigned to the new *spa* type t19075.

### 3.2. Antimicrobial Susceptibility Testing

The strains resulted sensible to all the antimicrobials tested in this study except for enrofloxacin for which all the strains were resistant at a concentration of ≤0.5 mg/L. The strains ProNaCC1 and ProNaCC6 resulted resistant for benzylpenicillin at a concentration of ≥0.5 mg/L (Table 4). The MIC values for all the antimicrobials tested in this study are included in the Supplementary Material (Table S11). 

### 3.3. General Features of Enterotoxigenic S. aureus 

The six enterotoxigenic *S. aureus* strains were whole-genome sequenced, and the draft assemblies ranged in size from 2.2 to 2.8 Mb with a range of 2126 to 2747 CDs (Table 5). 

**Figure 1:**
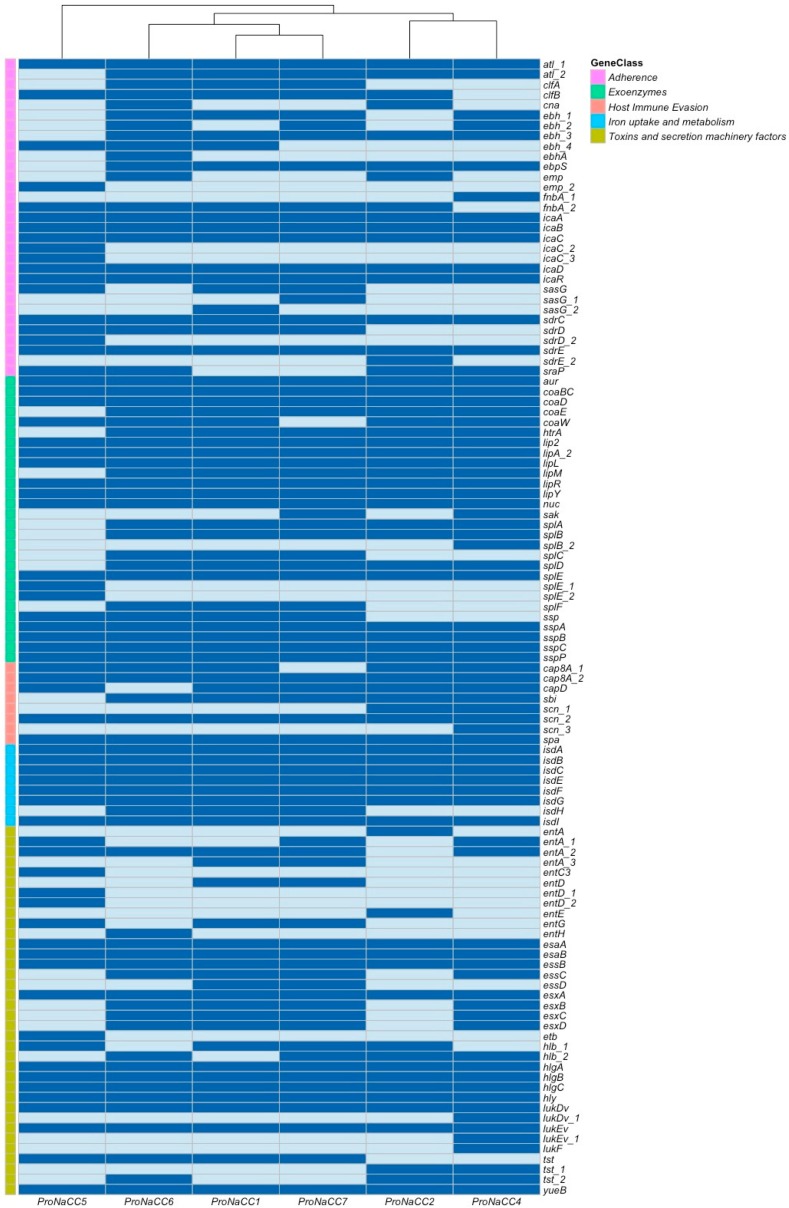
Heat map showing the profile of virulence factors (VFs) genes for the five enterotoxigenic *S. aureus* strains (dark blue: sequence detected; light blue: sequence not detected).

The genomes were analysed for the virulence factors and, 111 unique genes were identified and classified into five categories: adherence (n = 31), exoenzymes (n = 29), genes involved in host immune system evasion (n = 7); iron uptake regulatory system (n = 8); secretion machinery factors and gene of toxins (n = 36) (Figure 1).

#### 3.3.1. Adherence Factors

**Figure 2:**
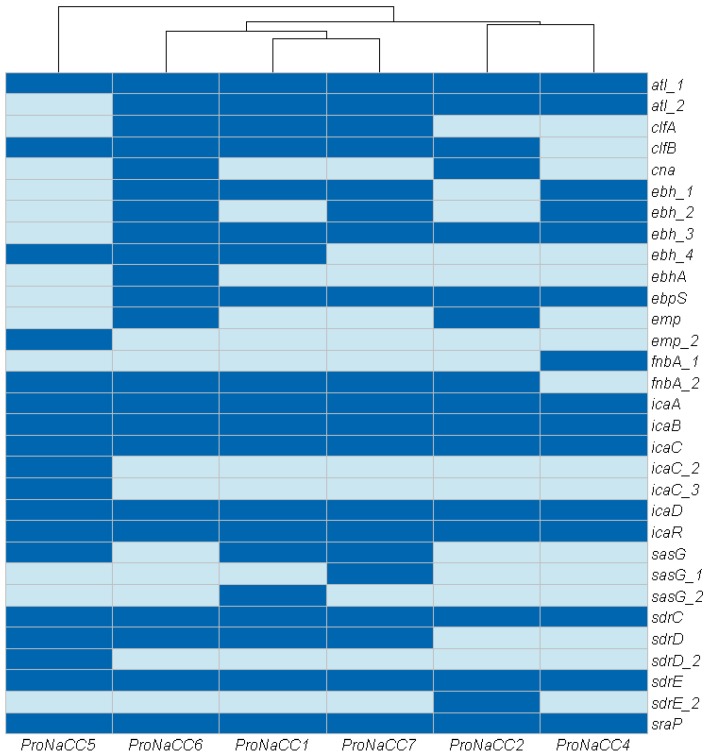
Heat map showing the adherence factors distribution among the six enterotoxigenic *S. aureus* strains (dark blue: sequence detected; light blue: sequence not detected).

In this study, 31 VFs were annotated in the genome of the six enterotoxigenic *S. aureus* strains related to adherence factors. The genes identified in this category were: autolysin (*atl*), genes involved in binding proteins and adhesion (*cna*, *ebh*, *ebpS*, *emp*, *fnbA*, *icaA*, *icaB*, *icaC*, *icaD*, *icaR*, *sdrC*, *sdrD*, *sdrE*, *sdrI* and *sraP*), clumping factor genes (*clfA*, *clfB*), and cell wall surface anchor protein (*sasG*) (Figure 2).

The genes *atl*, *icaA-C*, *D*, *R*, and *sdrC*, *E* were detected in all the strains; all the strains have at least one gene associated to bacterial proteins with the function of binding the major components of the extracellular matrix (*ebh*, *ebhA*, *fnbA*). The gene *cna* coding for collagen adhesin was detected only in the strains ProNaCC2 and ProNaCC6.

#### 3.3.2. *S. aureus* Exoenzymes

**Figure 3:**
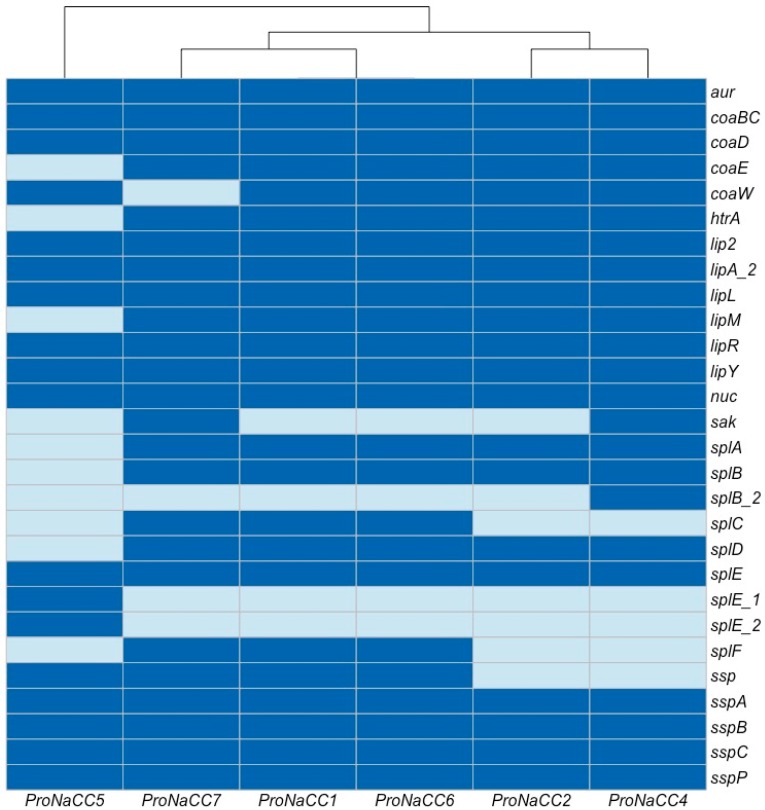
Heat map showing the exoenzymes genes among the six enterotoxigenic *S. aureus* strains (dark blue: sequence detected; light blue: sequence not detected).

In this class of VFs, 28 different genes were annotated, including the zinc metalloproteinase aureolysin (*coa*), serine protease family (*splA-F*), staphylokinase (*sak*), and other genes coding for enzymes (Figure 3).

All the genes coding for extracellular proteases were found in the six isolates, except for *splD,* which was not highlighted in the genome of the strain ProNaCC5 and *splF* on strains ProNaCC2, ProNaCC4 and ProNaCC5. All the other genes and at least one variant were found among all the genomes of the strain. Both the zinc metalloproteinase aureolysin (*aur*) and the thermonuclease (*nuc*) genes were found in all the strains.

#### 3.3.3. Genes Involved in Host Immune System Evasion 

**Figure 4:**
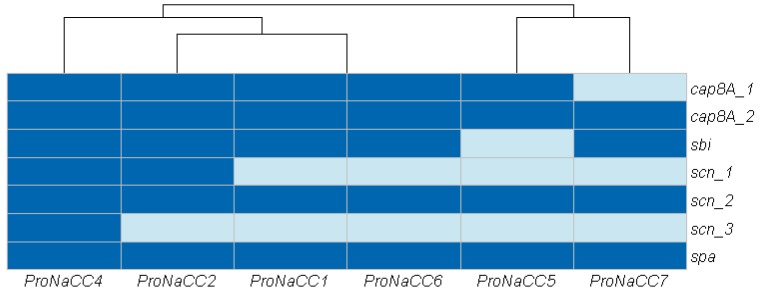
Heat map showing the genes involved in host immune system evasion among the six enterotoxigenic *S. aureus* strains (dark blue: sequence detected; light blue: sequence not detected).

In total, were detected seven genes of factors involved in host immune system evasion were detected, including the capsular synthesis (*cap8A_1 and 2*), immunoglobulin-binding protein (*sbi*) immunoglobulin G-binding protein A (*spa*) and the staphylococcal complement inhibitor (*scn*) (Figure 4).

The genes *cap* (*cap8A_1-2*) and *scn* (*scn_1-3*) and *spa* were detected on all the strains. The gene *sbi* was found, except in the strain ProNaCC5.

#### 3.3.4. Iron Uptake Regulatory System and Metabolism

**Figure 5:**
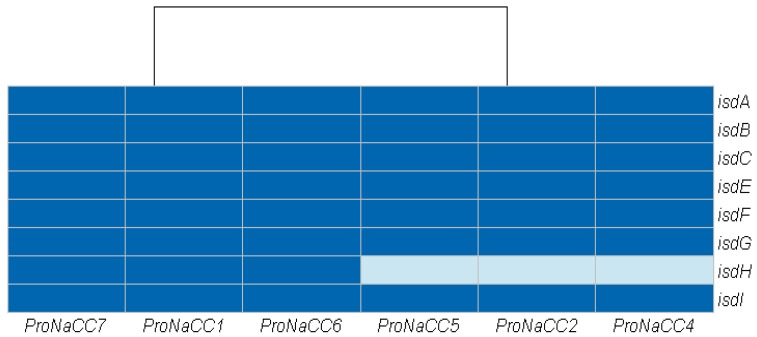
Heat map showing the genes coding for factors involved in the uptake of iron and metabolism among the six enterotoxigenic *S. aureus* strains (dark blue: sequence detected; light blue: sequence not detected).

In this class, eight genes coding for iron-regulated surface determinant proteins (*isdA*, *isdB*, *isdC*, *isdE*, *isdF*, *isdG*, *isdH,* and *isdI*) were identified (Figure 5).

The gene *isdH* is involved in the expression of the cell surface receptor IsdH for haemoglobin-haptoglobin complexes and was found in the genome of the strain ProNaCC1, ProNaCC6, and ProNaCC7; all the other genes were found in the genomes of the six strains.

#### 3.3.5. Toxins and Secretion Machinery Factors

**Figure 6:**
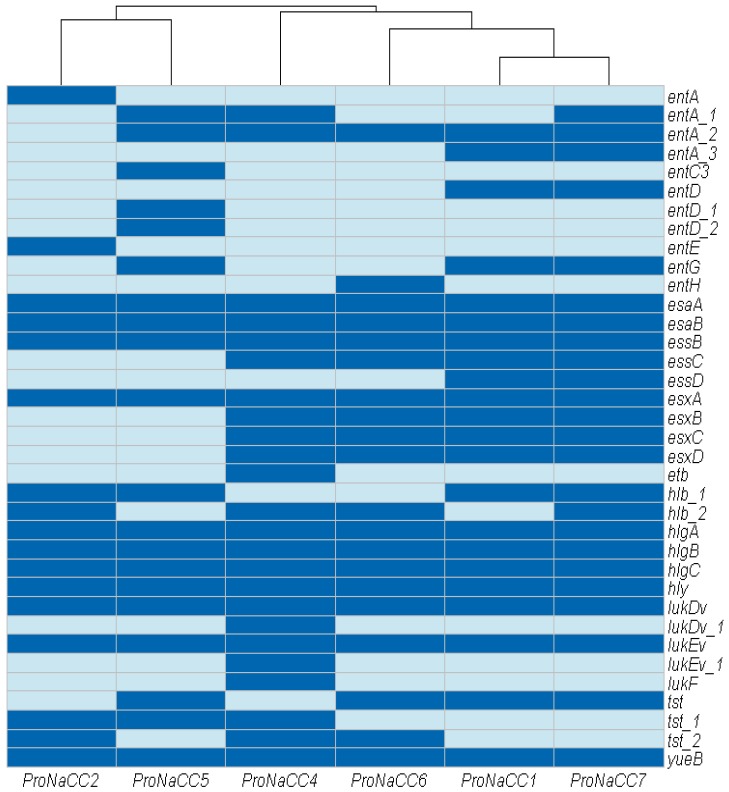
Heat map showing the genes coding for toxins and secretion machinery factors among the six enterotoxigenic *S. aureus* strains (dark blue: sequence detected; light blue: sequence not detected).

In this class the genes for toxins with haemolytic (*hlb*, *hlg component A-C*, *hly*) and leucotoxic activities (*lukEv*, *lukDv*, *lukF*), the genes involved in the secretion machinery (*esaA*/*esxA/yueB*, *esaB*/*esxB*/*essB*, *essC*/*esxC*, *essD*/*esxD*), the toxic shock syndrome toxin (*tst*), the exfoliative toxin type b (*etb*) and enterotoxins (*entA*, *entC*, *entD*, *entE*, *entG*, *entH*) were detected (Figure 6).

All the genes coding for the secretion machinery were detected in the strains ProNaCC1, ProNaCC6 and ProNaCC7. The strains ProNaCC6 and ProNaCC4 had all the genes for the secretion machinery, except the nuclease effector (*essD*) [70], the strains ProNaCC2 and ProNaCC5 had all except for the genes *essD* and *essC,* which are required for the secretion of substrates including *EsxA* and *EsxB* [71]. Among hemolysin genes, γ-hemolysins (*hlgA*, *hlgB*, *hlgC* and *hly*) were identified in all the strains, including the β-hemolysin (*hlb_1*) which was found in all the strains except in the strain ProNaCC4 and ProNaCC6 where it was found the gene *hlb_2* was found, which is the coding gene for a truncated protein. Different genes related to components of Leucotoxins were found in all the strains, in particular, the genes *lukEV* and *lukDV* coding for the Leucotoxin LukEv and LukDV, respectively. 

All the strains had at least one gene coding for enterotoxins either with intact sequences (*entA*, *entD*, *entE*, *entG*, *entH*) or for protein precursors (*entA_1*, *entA_2*, *entA_3*, *entD_1*, *entD_2*) and the exfoliative toxin type B (*etb*) was detected only in the strain ProNaCC4.

#### 3.3.6. Transcriptional Regulatory Elements

**Figure 7:**
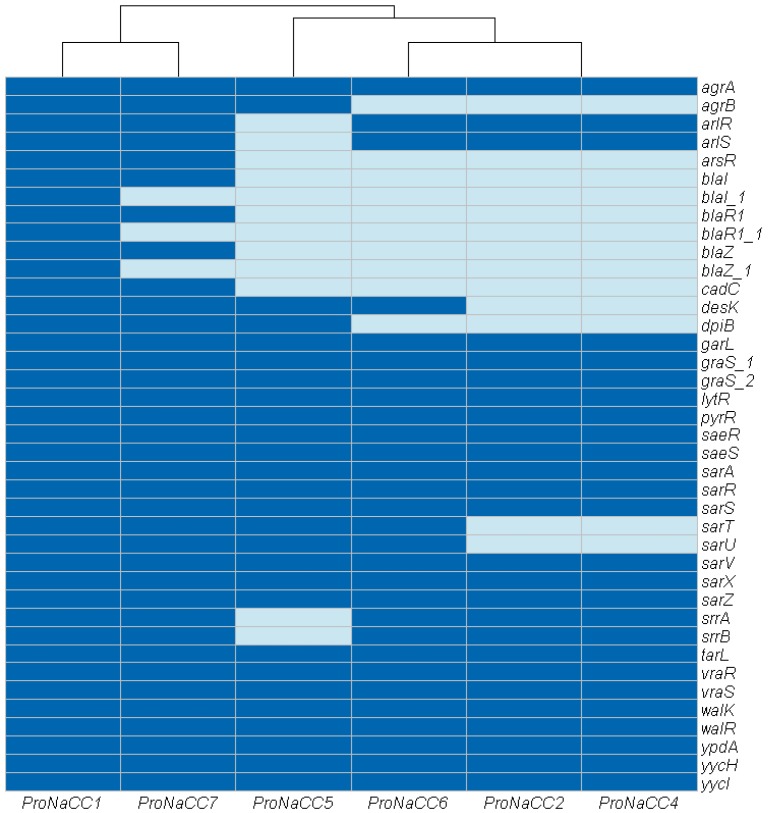
Heat map showing the genes coding for transcriptional regulator factors among the six enterotoxigenic *S. aureus* strains (dark blue: sequence detected; light blue: sequence not detected).

The genes involved in the production of transcriptional regulatory elements (n = 39) were grouped in a sixth group (Figure 7). 

In this category, a total of 39 genes were identified and, selected for their involvement as transcriptional regulators of staphylococcal virulence factors the genes *agrA*, *garL*, *graS_1*, *graS_2*, *lytR*, *pyrR*, *saeR*, *saeS*, *sarA*, *sarR*, *sarS*, *sarV*, *sarX*, *sarZ*, *srrA*, *srrB tarL*, *vraR*, *vraS walk*, *walR*, *ypdA*, *yycHm* and *yycI* were detected in all the genomes. The genes involved in the arsenical resistance operon repressor (*arsR*) were detected in the strains ProNaCC1 and ProNaCC7, and cadmium resistance transcriptional regulatory protein (*cadC*) was detected in the strains ProNaCC1, ProNaCC5, ProNaCC6, and ProNaCC7, and the transcriptional repressor that constitutively blocks the expression of β-lactamase, *bla* family (genes *blaI*, *blaI_1 blaR1*, *blaR_1 blaZ*, *blaZ_1*) was detected in the strains ProNaCC1 and ProNaCC7. The genes coding the *S. aureus* two-component systems (*srrA*-*srrB* and *arlR-arlS*) involved in the global regulation of VFs, were detected in all the strains except in the genome of the strain ProNaCC5.

### 3.4. Enterotoxin Insight

Further analyses were performed for the gene coding staphylococcal enterotoxins. A novel database was built for ABRicate starting from amino acid sequences extracted from GenBank nucleotide and uniprot databases [72]. The sequences manually annotated (Swiss-prot) and un-reviewed enterotoxin variants (TrEMBL) were translated with the EMBOSS tool [73] and combined to build the final database (Supplementary Material Database S3), which included, in total, 99 sequences of staphylococcal enterotoxins and their variants. The strain ProNaCC1 was positive for the genes of the enterotoxins SED, SElJ, SER, SelX, and SElW; the strains ProNaCC2, ProNaCC4, and ProNaCC6 were positive for both the gene of the enterotoxins SElX and SElW and for the genes of SEE, SEA, and SEH respectively. The strain ProNaCC5 was positive for the genes of SEY, SElX, SEG, SEN, SElU, SEI, SEM, and SEO, and the strain ProNaCC7 was positive for the gene coding for SED, SElJ, SER, SEA, SelW, and SElX (Table 6). 

The genomes were submitted to the PHASTER web server for the identification of prophage sequences and the output was tested for the presence of SEs with the database developed for ABRicate. The intact prophages regions and the hypothetical proteins sequences associated with prophages were considered in the study for the analysis of SEs similarities. According to the score assigned by the software, 20 of the prophages most common regions within the six enterotoxigenic genomes were identified: three regions resulted intact, 13 regions were incomplete, and four were questionable (Table S4).

The intact prophage most common regions were identified on the strain ProNaCC2 (ФN315, ФSa119), ProNaCC4 (ФN315, ФBU01), and ProNaCC7 (ФN315, ФSa119, ФNM3, ФBU01). The phage regions included the gene for the staphylococcal enterotoxin type E in the strain ProNaCC2 and the gene for the staphylococcal enterotoxin type A in the strains ProNaCC4 and ProNaCC7 (Table 7).

The genetic surrounding of the *tst* locus was further investigated for the genomic localisation, because this virulence factor can be associated with SaPIs, which includes the genes for specific staphylococcal enterotoxins. The database developed for this analysis was built for ABRicate and had sequences of 74 SaPIs included the specific sequences of SEs found in these loci (Supplementary Material Database S5). The analysis showed that all six strains contained the SaPI3 similar to the SaPI described in *Staphylococcus aureus* subsp. *aureus* COL [74] with a 100% coverage for specific genomic portions with an identity at the nucleotide level between 94.2% and 100% to the reference sequence. The other SaPIs with an identity percentage higher than 95.0 had a percentage of coverage less than or equal to 48.07 at the nucleotide level (Table S6). The SaPI1 (ref U93688.2) was identified in the genome of the strain ProNaCC2 with a percentage of coverage of 1.46 at the nucleotide level. A portion of the SaPI (fhuD) (ref AB983199.1) was found in the genome of PronaCC4 and ProNaCC5 with a coverage of 0.4% and 23.98%, respectively. The SaPIbov (ref. AF217235.1) was found in the genome of the strains ProNaCC4 with a percentage of coverage of 25.11 at the nucleotide level and at 0.44% in the genome of the strains ProNaCC5 and ProNaCC6. Those genomic loci were analysed for the detection of the SEs gene sequences, but intact sequences were not found (Table 7).

**Figure 8:**
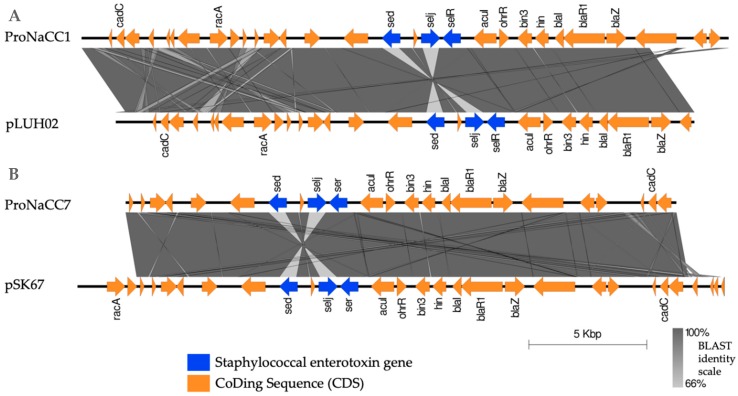
Comparison of plasmidic regions detected in the genome of the strains ProNaCC1 (**A**) and ProNaCC7 (**B**) with the plasmids pLUH02 and pSK67. The regions included the enterotoxin and enterotoxin-like genes and the coding sequences (CDS) detected in the regions.

In total, 4456 *S. aureus* plasmid complete sequences were used to build the novel ABRicate database (Supplementary Material Database S7). The database was filtered for the presence of staphylococcal enterotoxin sequences using the database developed in this study (Supplementary Material Database S3). The plasmids positive for SEs gene sequences were 186; from this group, the plasmids with the presence of enterotoxin genes with a percentage of coverage greater than or equal to 95.00 at the nucleotide level with the reference sequences were selected. In this selection, were found the gene sequences of the enterotoxin *sed*, *selj*, *ser*, *ses* and *set*, confirming the plasmid location of these genes [23]. The sequence of the plasmid pLUH02 [75] (ref. NZ_LJBK01000031.1) was found almost intact (98.11% coverage and 99.78% identity at the nucleotide level) in the genome of the strain ProNaCC1 (Table S8). The sequence of the plasmid pSK67 (ref. GQ900447.1) was found in the genome of the strain ProNaCC7 with an identity at the nucleotide level of 99.83% but a coverage lower than the threshold (85.14%). Both the plasmid sequences tested for the enterotoxin database resulted positive for the sequences of *sed*, *selj*, *ser* (Figure 8).

### 3.5. Antimicrobial Resistance Genes

**Figure 9:**
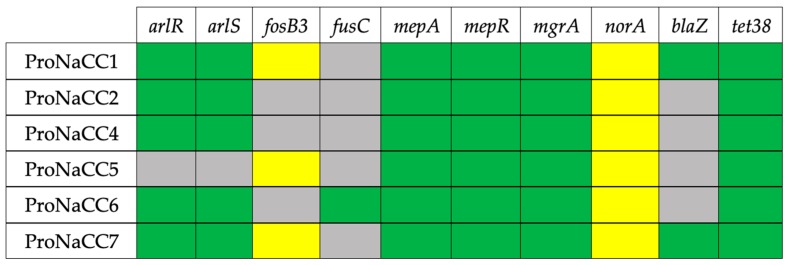
Genes related to antimicrobial resistance and their distribution among the six strains. The heatmap depicts the presence (green) or absence (grey) of genes. When mutations, such as internal deletions, were identified, they were highlighted in yellow. *arlR*: response regulator ArlR gene; *arlS*: signal transduction histidine-protein kinase ArlS gene; *fosB3*: metallothiol transferase FosB gene; *fusC*: fusidic acid resistance gene; *mepA*: multidrug resistance efflux protein MepA gene; *mepR* upstream repressor of *mepA* gene; *mgrA*: transcriptional regulator gene for *norA*, *norB*, and *tet38*; *norA*: quinolone resistance protein gene; *blaZ*: β-lactamase penicillin resistance gene; *tet38*: tetracycline efflux pump gene.

The transcriptional regulator gene *mgrA* was the only regulatory factor involved in multidrug resistance identified among the study strains. The gene was present in all six strains with sequence identity at the nucleotide level of 100% relative to the NC_002745 reference sequence (Figure 9). The gene *mgrA* is a regulator factor for *norA* and *tet38* which are the antibiotic resistance-encoding gene for quinolone and tetracycline, respectively [76], and were found in all six strains. The sequence identity at the nucleotide level of the gene *norA* was ranging between 76.01% and 76.23% relative to the AY566250 reference sequence. The gene *tet38* had a sequence identity at the nucleotide level ranging between 98.67% and 99.85% relative to the AY825285 reference sequence. In addition, the genes *arlR* and *arlS*, which are involved in the cascade reaction for the *norA* activation, were found in all the strains except the strain ProNaCC5. The penicillin resistance gene *blaZ* was identified in the strains ProNaCC1 and ProNaCC7. The gene was present in both the strains with a sequence identity at the nucleotide level of 96.45 relative to the CP000732 reference sequence. The fusidic acid resistance gene *fusC* was found only in the strain ProNaCC6 with a sequence identity of 100% with the NC_002953 reference sequence.

The lanthionine-containing peptide antibiotic (lantibiotic) gene (*bsaA2*) [77], was the only bacteriocin found among the six enterotoxigenic *S. aureus* genomes that had a sequence identity at the nucleotide level of 100% with the reference sequence of the bagel3 database. The sequence was found in the genome of the strains ProNaCC1, ProNaCC6, and ProNaCC7. The second highest percentage identity bacteriocin gene was the delta-hemolysin (*hld*), which had 80.00% of identity with the reference sequence, and it was found in the genome of ProNaCC1, ProNaCC4, ProNaCC5, and ProNaCC7. In total, 38 other bacteriocins (Table S9) with a sequence identity at the nucleotide level were found to range between 3.33% and 54.55%.

### 3.6. Core Genomes

**Figure 10:**
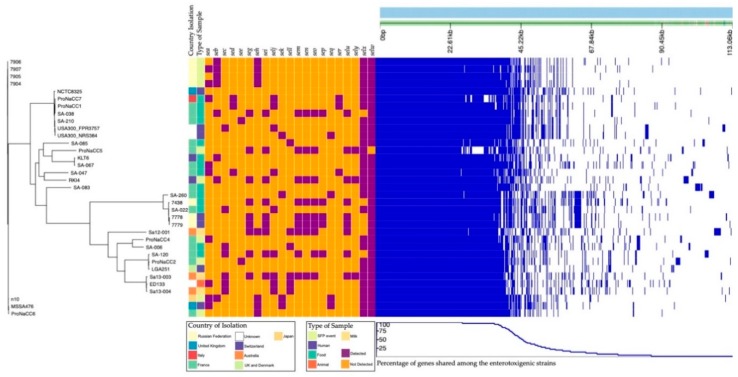
Pan-genomic analysis of the 35 enterotoxigenic strains. On the left side is the phylogenetic tree of the strains included in this study. On the central heatmap is the the geographical location of isolation and sources (colour legend in the picture). The detection of the gene coding for enterotoxins is reported as presence and absence with the colours purple and orange respectively.

A pan-genome of 5651 genes was generated from the six enterotoxigenic strains and 29 enterotoxigenic strains reported in the literature (Table S2). The total number of core genes was 1364, which represents the number of genes that are shared in a percentage higher than 99% among the six strains. Based on the output of Roary, insight into their pan-genomic property was also gained. In Figure 10, presents the tree constructed by clustering strains based on accessory genes and the matrix plot, which denotes the presence and absence of every gene over all strains by blue and white, respectively. Figure 10 presents the reported the geographic locations (Australia, Denmark, France, Italy, Japan, Russian Federation, Switzerland, United Kingdom), isolation sources (Staphylococcal Food Poisoning event, milk, clinical and food samples), and the enterotoxins identified with the ABRicate database for each strain. 

**Figure 11:**
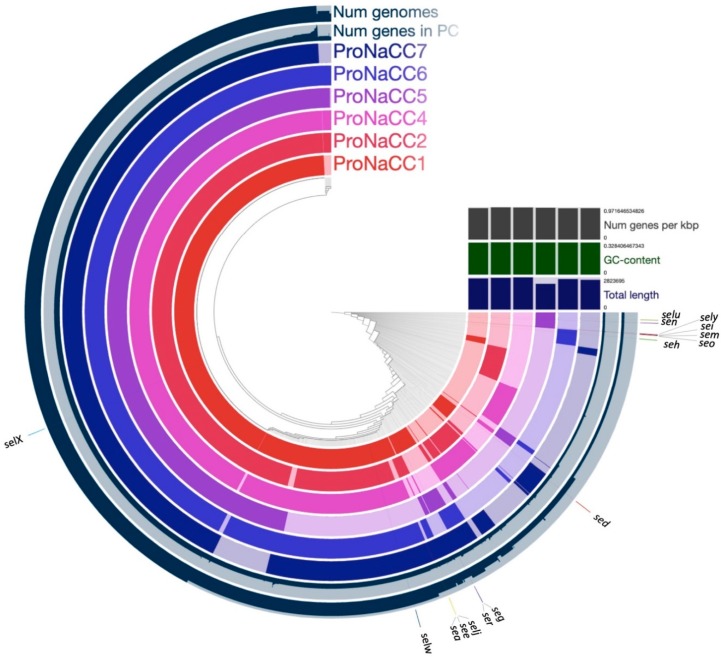
Clusters of Orthologous Groups (COG) function of the six enterotoxigenic *S. aureus* pan-genomes. The figure presents the location of the staphylococcal enterotoxin genes and their position. Num genomes: the bar represent the number of genomes per cluster; Num genes in PC: the graph represent the number of genes in protein clusters; GC-content: guanine-cytosine content. The genes of the enterotoxins are presented in the short form: *sea*, *sed*, *see*, *seg*, *she*, *sei*, *selj*, *sem*, *sen*, *seo*, *ser*, *selu*, *selw*, *selx*, *sely.*

At least two genes coding for staphylococcal enterotoxins were detected in all the genomes included in the analysis. The genes *selx* and *selw* were the most prevalent among the 39 strains; nevertheless, no correlation was found between the country of isolation and origin of the samples. The functional annotation of the six-enterotoxigenic *S. aureus* pan-genome was 66.2% of the COGs database with known functions of the total pan-genome (Figure 11). 

The pangenome of the six enterotoxigenic *S. aureus* was assembled to highlight the genetic and pathogenic diversity of the strains, and the locations of the SE genes compared to the core genome were validated. The gene of the staphylococcal enterotoxin-like x gene (*selx*) was detected in the core genome, as already described, along with the gene *selw*, in almost all the 35 strains included in the pangenome study. However, the genes coding for enterotoxins were detected mostly in specific accessory genomic regions, demonstrating the importance and the role of these genomic structures for the evolution of *S. aureus* as foodborne pathogens.

### 3.7. SEs Production and Naturally-Contaminated Cheeses

The strains ProNaCC1, ProNaCC2 and ProNaCC4 produced SED, SEE and SEA in 0.85% NaCl solution respectively. The strains ProNaCC5 and ProNaCC6 resulted negative for the production of toxins in vitro and the strain ProNaCC7 produced both SEA and SED. The milk used in this study had a fat content of 36.0 g per litre, 49.0 g of carbohydrates per litre, 32.0 g of proteins per litre, and 0.9 g of NaCl per litre as the average content declared by the producer. Four batches of the milk incubated with the enterotoxigenic strains (18 h) resulted positive at both qualitative methods for SEs detection (Table 8) for the enterotoxins detectable with the available methods. The ripened wheels were subsampled on peripheral (rind portion) and internal (core portion) sampling area and resulted positive to qualitative methods. The concentration of the toxins in the core ranged from 1.833 ng/g for the SEA for the batch number 6 (ProNaCC7) and 9.126 ng/g for toxin type E detected in cheese number 2 (inoculated with strain ProNaCC2); the concentration in the peripheral part (rind) ranged from 1.849 ng/g for SEA in cheese batch number 6 and 8.419 ng/g for SEE in cheese batch number 2.

The six batches of spiked milk resulted positive for CPS after 18 h of enrichment at 37 °C with a concentration between 2.1 × 10^7^ and 1.1 × 10^8^ CFU/g. The fresh cheeses were tested for CPS after 3 h of ripening (sweating stage) and their number was between 2.5 and 5.6 × 10^8^ CFU/g (Table 9).

## 4. Discussion

In this study, six enterotoxigenic strains of *S. aureus* were whole-genome sequenced and the distribution of 110 VFs and 39 genes involved in the production of transcriptional regulatory elements was determined. The strains resulted positive for the staphylococcal enterotoxins genes commonly reported as a causative agent of food poisoning and the associated computational analysis added a detailed characterisation and identification of further enterotoxin genes that complete the comprehensive portraits of the strains presented. Previous studies reported food poisoning outbreaks caused by enterotoxigenic *S. aureus*, and they were focused on the outbreak investigation, strain genotyping and characterisation of produced SEs [25,27,28,29,78,79]. Other studies included the identification of different VFs of *S. aureus* enterotoxigenic strains isolated from foods and from SFP outbreaks by PCR [80,81] with the use of microarray-based methods [82] and analysing the whole genome sequences [83,84]. However, these studies focused on the presence of few VFs or specific staphylococci related to host [85]. In this study, 149 genes were detected in the genome of the enterotoxigenic strains and the genes related to staphylococcal enterotoxins were further characterised with the use of dedicated databases developed in this study. The VFs were grouped into six functional categories and their distribution was determined in the six enterotoxigenic strains. In addition, the strains with other 29 enterotoxigenic strains reported in the literature were used for constructing the pan-genome of enterotoxigenic *S. aureus*, highlighting the genomic location of the enterotoxins and their role in the evolution of its genome.

The virulence of *S. aureus* is facilitated by the attachment to the host cell and is mostly mediated by adhesins [86]. In the genome of the six enterotoxigenic *S. aureus* strains 31 genes coding for proteins that mediate the adherence to damaged tissues, extra-cellular matrix, and the surface of cells were annotated. The intercellular adhesion (*ica*) locus consists of different genes (*icaA*, *icaB*, *icaC*, *icaD*) and the promotor *icaR,* which are involved in the biofilm formation initiated by *atl* [87,88]. In other studies, the strains *ica*-positive were isolated in both the food and clinical environment, but their presence was not always associated with the production of biofilm [89]. The database included other genes involved in the formation of biofilms, i.e., the accumulation-associated protein gene (*aap*) and the biofilm-associated protein (*bap*), but none of the six enterotoxigenic strains resulted positive. Other genes associated with adhesion, invasion, and evasion were found in all the strains, in particular, the genes *sdrC* and *sdrE*, which were reported in clinical isolates [90,91].

Enterotoxigenic *S. aureus* can produce exoenzymes which are active against the immune system and have a proteolytic effect on complex molecules. The genes coding for thermonuclease (*nuc*), aureolysin (*aur*), staphylocoagulase (*coa*), lipase (*lip*), and extracellular matrix protein-binding protein (*ssp*) were found in all the strains; this result was demonstrated in other studies for non-*aureus* staphylococci, where these genes were the most reported among this group of VFs [86,92]. The gene for HtrA surface protease was found in all the strains except the strain ProNaCC5. The protein is involved in the virulence of many pathogens, and it has a role in stress resistance and bacterial survival [93]. The genes coding for serine protease-like proteins (*splA-F*) are organized in an operon and exhibit proteolytic activity [94]. At least one gene of these exoenzymes was found in all the strains and have not been described in enterotoxigenic *S. aureus* isolates yet. Only the strains ProNaCC4 and ProNaCC7 had the gene for staphylokinase (also known as SAK) (*sak*). This virulence factor is located in different prophages, including the prophages carrying the genes for the enterotoxin SEA [23], which was found in both the strains.

The virulence of *S. aureus* is related to the ability to evade the host immune system and to produce capsular polysaccharides, including the enterotoxigenic strains [95]. Among this class, the genes located in the immune evasion cluster (*sbi*, *scn* and *spa*) were detected; the presence of these genes can be an advantage for pathogenesis of these enterotoxigenic strains as suggested in different studies where the genes were considered highly specific for staphylococcal isolates of human origin, including methicillin-resistant and methicillin-sensitive *S. aureus* [96].

The VFs involved in the uptake of iron and metabolism found among the six enterotoxigenic strains were part of the iron-regulated surface determinants (*isd*). This group of genes is encoded by five transcriptional units including *isdA*, *isdB*, *isdC-D-E-F-srtB-isdG*, *isdH*, and *isdI* [97], which were found in all the six enterotoxigenic strains except the gene *isdH* that was not detected in the strains ProNaCC2, ProNaCC4 and ProNaCC5. The accessory gene regulator *agr* is a *S. aureus* quorum-sensing system involved in the control of different VFs including toxins [98]. This system is a multicomponent signal transduction system that is able to regulate the enterotoxin production through a complex network of pathways [99,100]. The gene *agr* was detected in all the strains within the genes for SarA protein family, SrrAB protein, and SaeRS two-component system that controls the expression of several virulence factors with both positive and negative effects. The genes coding for the member of the two-component regulatory system WalK/WalR, VraS/VraR and the genes of the YycH family protein were found in all the six enterotoxigenic strains. These factors are involved in the control of autolysis, biofilm formation and cell wall metabolism [101].

The toxins produced by *S. aureus* are different molecules that can be grouped in hemolysins, leukocidins/leukotoxins, toxic shock syndrome toxin, exfoliative toxins, exotoxins and enterotoxins. In addition, the genes involved in the secretion system were included in this group of genes and all the six strains contained at least one of them (*esaA*/*esxA*/*yueB*, *esaB*/*esxB*/*essB*, *essC*/*esxC*, *essD*/*esxD*) previously described as a versatile secretion system in *S. aureus* that might be involved in the transport of protein and or DNA [71]. The strains resulted positive for the staphylococcal enterotoxins genes commonly reported as a causative agent of food poisoning. With the use of the novel ABRicate database for enterotoxins and their variants, further enterotoxin genes were identified, which completed the comprehensive portraits of the strains presented in this study. The genomes were used to construct the pangenome of enterotoxigenic *S. aureus*, and the enterotoxin *selX* was detected as a component of the core genome [34]. The gene for this toxin is encoded in the core genome of 95% of phylogenetically diverse *S. aureus* strains, according to a study that included strains isolated in the case of human and animal infections [102]. Other enterotoxins were detected in mobile genetic elements, in particular, the prophage regions coding for the enterotoxin type E (ФN315 and ФSa119) that were found in the strain ProNaCC2 and the gene for the staphylococcal enterotoxin type A, which was detected in the strain ProNaCC4 (ФN315 and ФBU01 prophage sequences) and ProNaCC7 (ФN315, ФSa119, ФNM3 and ФBU01 prophage sequences), suggesting the phagic origin of these enterotoxins [23]. *S. aureus* acquires VFs encoded in the mobile genetic elements such as prophages but also plasmids, pathogenicity, and genomic islands, providing adaptation but also a genetic rearrangement resulting in hypervirulence of some strains but also the loss of portion of genes [44]. Interestingly, the sequences *entA*_*1-3* were found in the genome of all the strains (Figure 6). These sequences are classified as enterotoxin family protein elements but they are not complete or have a low percentage of coverage and identity at the nucleotide level to the reference sequence of the enterotoxin type A. For this reason, the sequences of SEA genes were not confirmed with the database ABRicate for enterotoxins, confirming the importance of a validated database for staphylococcal enterotoxins that considers the manual validation of sequences.

The SaPIs database was used for evaluating the presence of enterotoxins in these genetic structures and at least one SaPI was found in each strain showing that SaPIs are widespread among the six enterotoxigenic strains. The sequences did not carry out SE genes but identical sequences among the strains were detected, also in the form of small fragments, explaining their phylogenetic relationship due probably to horizontal transfer.

The genes for the plasmid encoded enterotoxins SED, SElJ and SER were found in the genome of the strains ProNaCC1 and ProNaCC7. The plasmid sequences found in the genome of the strain ProNaCC1 had a 98.11% coverage and 99.78% identity at the nucleotide level with the plasmid pLUH02 (accession number NZ_LJBK01000031.1) (Table S8), which was described in a strain responsible for two outbreaks in a maternity hospital in Belgorod, Russia [75]. The sequences of the *S. aureus* plasmid pSK67 (accession number GQ900447.1) were identified in the genome of the strain ProNaCC7 and had an 85.14% of coverage and 99.83% identity with the reference sequence (GQ900447.1) (Table S8). The gene coding enterotoxins (*sed*, *selj*, *ser*) were found within the sequences of the plasmids confirming their origin from mobile genetic elements. 

The sequences of the plasmids detected in this study had similarity at nucleotide identity between 98.87% and 99.96% with the plasmids pIB485 (accession number AF053140.1 and M94872.1) and pF5 (accession number AB330135.1) reported in strains responsible for food poisoning [23,103,104]. However, the coverage corresponds only for the sequences of *sed* and *selj*, which are carried out in pIB485 and the gene *selj* and *ser* carried out in pF5, while both the strains (ProNaCC1 and ProNaCC7) carried out the sequences of the genes *sed*, *selj,* and *ser* in a unique putative plasmid region. In addition, the plasmid encoded genes involved in the arsenical resistance operon repressor (*arsR*) was detected in the two strains (Figure 8) along with the gene *blaZ* (Figure 9), responsible for the resistance to benzylpenicillin. The gene encoding the production of β-lactamases can be located in the chromosome, in plasmids, or in plasmid integrated into chromosome and transposon integrated into chromosome [105] and in these strains, the gene *blaZ* was detected within the plasmidic-origin enterotoxins, confirming the presence of the two plasmids, pLUH02 and pSK67, respectively, in the strain ProNaCC1 and ProNaCC7.

The strain ProNaCC5 resulted positive for several non-classical staphylococcal enterotoxin genes (*seg*, *sei*, *sem*, *sen*, and *seo*) and the staphylococcal enterotoxin-like genes (*selu*, *selx*, and *sely*). In Table 5, the position of start and end of the sequences within the contigs of the SEs genes was reported, and the genomic structure of the enterotoxin gene cluster which includes the genes for the enterotoxins SEG, SEI, SEM, SEN, SEO, and SElU was demonstrated [106]. The strain was isolated in food suspected as responsible for SFP, but the method for the detection of enterotoxins did not identify any of the classical SEs. A strain with the same gene cluster, was responsible for an outbreak in Osaka city, Japan [107]. Unfortunately, also in this SFP, it was not possible to identify any classical enterotoxin in the food, similarly to the result obtained with the naturally-contaminated cheese, where the absence of enterotoxin SEA-SEE was confirmed (Table 6). Different studies revealed the isolation of staphylococcal strains implicated in SFP outbreaks in which classical SE genes were not detected, harbouring one or more of the new se/se-like genes, i.e., *seg*, *seh*, *sei*, or *selj* [37,108,109], and their toxins might have been the cause of these outbreaks. The genomic island vSaβ type III, contains the genes for serine proteases (*splC*, *splE* and *splF*) in addition to the SEs/SEl genes [23], but the strain ProNaCC5 resulted positive only for the gene *splE*, suggesting a possible variant of this genomic island.

The penicillinase encoded by *blaZ* was found in the strains ProNaCC1 and ProNaCC7, as previously reported. In this study, the gene was found in the plasmids pLUH02 and pSK67, detected in the strains ProNaCC1 and ProNaCC7, respectively. The strain ProNaCC1 resulted resistant to the antibiotic benzylpenicillin (≥0.5 g/L), suggesting a role of the gene in the resistance mechanism. On the other hand, the strain ProNaCC7 resulted sensible at low concentrations of the antibiotic (0.06 g/L) (Table S11), and the strains ProNaCC6 resulted resistant (≥0.5 g/L). The gene of the *S. aureus* fluoroquinolone efflux transporter protein NorA was detected in all the sequenced strains. The overexpression of this gene causes the resistance mechanism, but unfortunately transcriptional data were not included in this study. Nevertheless, all the strains resulted resistant to enrofloxacin at a concentration of ≤0.5 g/L.

The pangenome of the six enterotoxigenic *S. aureus* was assembled and applied to highlight the genetic, metabolic, and pathogenic diversity of the species in comparison with other 29 enterotoxigenic strains. Consistent with prior studies, the numbers of new genes per genome indicated substantial intraspecific variation and overall pangenome “openness” associated with a broad niche range [110,111] but it is also a characteristic of populations that undergo frequent horizontal gene transfer [112]. In fact, *S. aureus* is a versatile microbe in complex microbial communities both in the environment or in a specific host, shifting between commensal or pathogenic variant [113,114]. Functional assignment of the core genes revealed that they are mostly associated with housekeeping functions (i.e., control of gene expression machinery and basic biochemistry). This confirms previous observations that *S. aureus* is a clonal species [115]. Recent studies have shown that even mutations in the core genome of closely related *S. aureus* isolates can have significant effects on virulence, proliferation, and persistence [116]. Thus, comparative studies are necessary to find these possible changes, and even more so, in enterotoxigenic strains that might be involved in food contaminants and foodborne outbreaks. A particular emphasis was placed on the analysis and distribution of enterotoxin genes among the six enterotoxigenic strains (Figure 11) and, the gene coding for the novel SAg staphylococcal enterotoxin-like toxin X (gene *selx*) was found in the core genome of the six enterotoxigenic strains; this result is comparable with another study where the gene was found in the core genome of 95% of a diverse *S. aureus* population, including isolates from human and animal infections [102]. The other genes coding for staphylococcal enterotoxins were detected onto mobile genetic elements (Table 7) and is one of the reasons why the pangenome resulted closed. In fact, the addition of other enterotoxigenic strains in the dataset does not provide new genes to the species pangenome. This feature is typical for species that live in isolated niches with limited access to the global microbial gene pool; indeed, the genomic and phenotypical diversity is due to the transfer of genetic material, including resistance genes and enterotoxins. Despite this, all the strains showed substantial differences regarding the accessory genes. Indeed, the six genomes we characterised by a highly dynamic mobilome (gene clusters, phage-related enterotoxins, SaPIs, and plasmids). In this study, 29 *S. aureus* genomes of isolates that were considered enterotoxin-producers in addition to reference strains used for the genome comparison were included. Interestingly, it was possible to identify other genes coding for enterotoxins in addition to the genes that were reported in the literature, especially novel enterotoxins that were not genetically characterized at the time of the publication of the sequences. This result underlines the importance of a novel bioinformatics approach for typing the enterotoxins which can be produced by *S. aureus*, and their use as a tool in support of diagnostics.

As demonstrated in different studies, food-related stress conditions, such as NaCl, nitrite, glucose and lactic acid variations encountered during food production and preservation, can induce significant changes in the activity of SEB and SED promoter genes [117,118]. In this study six batches of cheese naturally contaminated with *S. aureus* strains were produced. The cheesemaking, followed a protocol that was published before by Bianchi et al. [119] for the production of naturally contaminated cheese with the enterotoxin type D, and the parameters that would have altered the enterotoxigenic *S. aureus* cells or their survival during the ripening were not recorded. The enumeration of coagulase positive staphylococci (CPS) resulted high after the sweating process, between 2.5 and 5.6 × 10^8^ CFU/g. In fact, according to the EC Regulation 2073/2005 on microbiological criteria for foodstuffs, when CPS enumeration exceeds 10^5^ CFU/g or CFU/mL, the samples must be tested for enterotoxins due to their presumed presence if the CPS population is constituted by enterotoxigenic strains. SEs detection methods rely on commercially available polyvalent enzyme-linked immunoassays (ELISA) or enzyme-linked fluorescent immunoassay (ELFA) with antibodies that detect SEA-SEE and the cheese samples spiked with the strain ProNaCC1-4 and ProNaCC7 resulted positive for at least one SE (Table 8). The strains ProNaCC5 and ProNaCC6 were isolated in cheese and in a composed salad, respectively (Table 1), and resulted both negative for the five detectable enterotoxins in the naturally-contaminated cheese study. Interestingly, the strain ProNaCC5 was isolated during a food poisoning investigation and were involved five patients with symptoms similar to staphylococcal food poisoning, and other pathogens in the foods were not isolated. From the genetic point of view the two strains are different; the strain ProNaCC6 resulted positive for the gene coding for the enterotoxin SEH, already reported as responsible for food-borne outbreaks and associated to a transposon [120,121] and the enterotoxin-like genes X (core genome) and W, while the strain ProNaCC5 resulted positive for the genes carrying out the enterotoxin-like type U, Y, and X (core genome) and the gene-cluster encoded enterotoxins (type G, I, M, N, O). The *egc* enterotoxins or a combination of them (SEG, SEI, SEM, SEN, SEO) could have caused the SFP as reported in previous studies [31] supporting the involvement of the gene-cluster encoded enterotoxins rather than the core-genome enterotoxin-like in the event of foodborne poisoning caused by staphylococcal enterotoxins.

This study was limited to the genomic and in-silico prediction of the presence of VFs and SEs. Future developments and validation of methods for the detection of not-classical SEs and proteomic approaches will allow for a better understanding of in vivo production of the enterotoxins. The strains were whole-genome sequenced and were detected virulence factors, antimicrobial resistance genes, and mobile genetic elements with a specific focus on the staphylococcal enterotoxin and enterotoxin-like genes. Using novel databases offers a comprehensive portrait of enterotoxigenic strains of *S. aureus* used for the production of naturally-contaminated cheeses, and in the future, the identification of SEs and the development of innovative computational approaches will allow more accurate identification of additional genetic factors and genes necessary for the production of enterotoxins in food.

## 5. Data Access

The data have been deposited as a BioProject PRJNA419794 in the National Center for Biotechnology Information. The SRA are available at the following accession numbers: SRX3422350, SRX3422349, SRX3422348, SRX3422347, SRX3422346, SRX3422345.

## Figures and Tables

**Table 1:** Origin and details of the strains selected for this study.

Strain	Origin	Year	City, Country	Etiological Food	Patients	Symptoms ^1^	Reference
ProNaCC1	Routine analysis	2009	Haute-Savoie, France	Cheese (Tomme)	Not involved	Not reported	This study
ProNaCC2	Staphylococcal Food-borne Poisoning	2009	Sommes, France	Cheese (Mont d’Or du Jura)	23	AC, D, F, N, V	[47]
ProNaCC4	Staphylococcal Food-borne Poisoning	2012	Hautes-Vienne, France	Cheese (Basque Lait Cru Brebis)	3	AC, D, N, V	This study
ProNaCC5	Staphylococcal Food-borne Poisoning	2014	Loire, France	Cheese (Raclette)	5	AC, D, V	This study
ProNaCC6	Routine analysis	2014	Alpes de Haute Provence, France	Composed Salad (tuna-corn-beets-apple)	Not involved	Not reported	This study
ProNaCC7	Routine analysis	2015	Vercelli, Italy	Cheese (Tomme)	Not involved	Not reported	[48]

^1^ Detailed information about the symptoms reported: AC: Abdominal cramps; D: diarrhoea; F: Fever; N: Nausea; V: Vomiting.

**Table 2:** Primers used for this study. The amplification conditions were the same for both the first mPCR (*sea* to *see* and *ser*) and second mPCR (*seg* to *selj* and *ser*), except the annealing temperature (respectively 55 °C and 52 °C): initial denaturation 94 °C for 3 min; 35 cycles of denaturation (94 °C for 30 s), annealing (30 s), extension (72 °C for 90 s); final extension 72 °C for 7 min.

Gene	Name Primer	Sequence (5′-3′)	Product Size (bp)	Reference
*sea*	GSEAR-1	GGT TAT CAA TGT GCG GGT GG	102	[53]
	GSEAR-2	CGG CAC TTT TTT CTC TTC GG		
*seb*	GSEBR-1	GTA TGG TGG TGT AAC TGA GC	164	[53]
	GSEBR-2	CCA AAT AGT GAC GAG TTA GG		
*sec*	GSECR-1	AGA TGA AGT AGT TGA TGT GTA TGG	451	[53]
	GSECR-2	CAC ACT TTT AGA ATC AAC CG		
*sed*	GSEDR-1	CCA ATA ATA GGA GAA AAT AAA AG	278	[53]
	GSEDR-2	ATT GGT ATT TTT TTT CGT TC		
*see*	SA-U	TGT ATG TAT GGA GGT GTA AC	213	[53]
	SA-E rev	GCC AAA GCT GTC TGA G		
*ser*	SER 1	AGA TGT GTT TGG AAT ACC CTA T	123	[53]
	SER 2	CTA TCA GCT GTG GAG TGC AT		
*seg*	SEG-F	GTT AGA GGA GGT TTT ATG	198	[54]
	SEG-R	TTC CTT CAA CAG GTG GAG A		
*seh*	SEH-F	CAA CTG CTG ATT TAG CTC AG	173	[54]
	SEH-R	CCC AAA CAT TAG CAC CA		
*sei*	SEI-F	GGC CAC TTT ATC AGG ACA	328	[54]
	SEI-R	AAC TTA CAG GCA GTC CA		
*selj*	SEJ-F	GTT CTG GTG GTA AAC CA	131	[54]
	SEJ-R	GCG GAA CAA CAG TTC TGA		
*sep*	SEP-F	TCA AAA GAC ACC GCC AA	396	[54]
	SEP-R	ATT GTC CTT GAG CAC CA		

**Table 3:** Molecular typing of the strains.

Strain	Gene(s)	*Spa*-Type	MLST
*S. aureus* ProNaCC1	*sed*	t2953	ST-8
*S. aureus* ProNaCC2	*see*	t4461	ST-425
*S. aureus* ProNaCC4	*sea*	t19075 ^1^	ST-581
*S. aureus* ProNaCC5	*seg*/*sei*	t164	ST-389
*S. aureus* ProNaCC6	*seh*	t127	ST-1
*S. aureus* ProNaCC7	*sea*/*sed*/*selj*/*ser*	t3802	ST-8

^1^ The strain was identified as a new *spa*-type.

**Table 4:** Detected resistance to antimicrobials among the strains included in this study.

Strain	Antimicrobial	Class	MIC (mg/L)
ProNaCC1	Benzylpenicillin	Penicillins	≥0.5
ProNaCC1	Enrofloxacin	Fluoroquinolones	≤0.5
ProNaCC2	Enrofloxacin	Fluoroquinolones	≤0.5
ProNaCC4	Enrofloxacin	Fluoroquinolones	≤0.5
ProNaCC5	Enrofloxacin	Fluoroquinolones	≤0.5
ProNaCC6	Benzylpenicillin	Penicillins	≥0.5
ProNaCC6	Enrofloxacin	Fluoroquinolones	≤0.5
ProNaCC7	Enrofloxacin	Fluoroquinolones	≤0.5

**Table 5:** Feature of the six sequenced genomes assembled with SPAdes.

Feature	ProNaCC1	ProNaCC2	ProNaCC4	ProNaCC5	ProNaCC6	ProNaCC7
**Size (bp)**	2,728,931	2,782,905	2,823,695	2,260,169	2,677,370	2,607,693
**Contigs (>500 bp)**	19	45	54	15	9	14
**Contigs > 1 kb**	19	40	35	11	8	14
**CDs ^1^**	2570	26,701	2747	2126	2473	2463
**G/C content**	32.53%	32.80%	32.78%	32.71%	32.66%	32.61%

^1^ Coding sequences (CDs) assigned by PATRIC.

**Table 6:** Staphylococcal enterotoxins found with the use of the database for ABRicate.

Strain	Enterotoxin	Contig	Sequence Start ^1^	Sequence End ^2^	% Coverage ^3^	% Identity ^4^	Reference ^5^
ProNaCC1	SED	14	12,761	13,533	99.87	84.73	UniProtKB—R9SA89
	SElJ	14	14,428	15,212	97.64	83.95	UniProtKB—O85217
	SER	14	15,329	16,103	99.74	84.9	UniProtKB—Q76LS8
	SElX	3	249,399	250,007	100	84.24	UniProtKB—G0Z026
	SElW	5	93,584	94,369	100	100	GB—KX655710.1
ProNaCC2	SEE	1	409,445	410,214	99.87	83.9	GB—WP_044122767
	SElX	24	4093	4701	100	84.24	UniProtKB—G0Z026
	SElW	6	46,777	47,558	99.36	96.55	GB—KX655711.1|
ProNaCC4	SEA	1	983	1744	98.57	81.15	UniProtKB—P0A0L2
	SElW	3	200,805	201,585	99.36	96.16	GB—KX655711.1
	SElX	7	89,367	89,975	100	84.4	UniProtKB—G0Z026
ProNaCC5	SElY	2	58,778	59,440	100	84.77	UniProtKB—A0A0K2S2V0
	SElX	2	634,655	635,263	100	84.56	UniProtKB—G0Z025
	SEG	9	13,778	14,550	99.87	86.16	UniProtKB—P0A0L8
	SEN	9	14,836	15,609	100	85.92	UniProtKB—A0A0H3JS72
	SElU	9	15,630	16,397	98.08	84.42	UniProtKB—Q6XXM3
	SEI	9	16,560	17,279	99.17	87.5	UniProtKB—O85383
	SEM	9	17,320	18,033	99.58	85.01	UniProtKB—A0A0H3K005
	SEO	9	18,317	19,096	100	87.82	UniProtKB—A0A0H3JS76
ProNaCC6	SElW	1	1,068,149	1,068,933	99.87	97.84	GB—KX655711.1
	SElX	2	96,391	96,999	100	82.92	UniProtKB—G0Z026
	SEH	5	98,979	99,700	99.86	86.29	UniProtKB—P0A0M0
ProNaCC7	SED	11	6058	6830	99.87	84.73	UniProtKB—R9SA89
	SElJ	11	7725	8509	97.64	83.95	UniProtKB—O85217
	SER	11	8626	9400	99.74	−84.9	UniProtKB—Q76LS8
	SEA	2	88,812	89,579	99.61	85.81	UniProtKB—P0A0L2
	SElW	5	93,897	94,682	100	100	GB—KX655710.1
	SElX	7	241,512	242,120	100	84.24	UniProtKB—G0Z026

^1^ Sequence Start: start coordinate in the sequence; ^2^ Sequence End: end coordinate in the sequence; ^3^ % Coverage: proportion of gene covered; ^4^ % Identity: proportion of exact nucleotide matches; ^5^ Reference: the genomic source of the sequence (UniProtKB: UniProt Knowledgebase; GB: GenBank nucleotide reference number.

**Table 7:** Detection of mobile genetic elements and the presence of staphylococcal enterotoxins genes and percentage of coverage to the reference sequences.

Strain	SaPI1 ^1^	SaPI3 ^2^	SaPI3 ^3^	SaPI3 ^4^	SaPI3 ^5^	SaPI (fhuD) ^6^	SaPIbov ^7^	Prophage (Gene SE)	Plasmid pLUH02 ^8^	Plasmid pSK67 ^9^
**ProNaCC1**	n.d.	100%	100%	n.d.	n.d.	n.d.	n.d.	n.d.	98.11% (*sed*, *selj ser*)	n.d.
**ProNaCC2**	1.46%	100%	n.d.	48.07%	100%	n.d.	n.d.	ФN315, ФSa119 (*see*)	n.d.	n.d.
**ProNaCC4**	n.d.	n.d.	n.d.	n.d.	100%	0.40%	25.11%	ФN315, ФBU01 (*sea*)	n.d.	n.d.
**ProNaCC5**	n.d.	100%	n.d.	n.d.	n.d.	23.98%	0.44%	n.d.	n.d.	n.d.
**ProNaCC6**	n.d.	100%	100%	n.d.	n.d.	n.d.	0.44%	n.d.	n.d.	n.d.
**ProNaCC7**	n.d.	100%	100%	n.d.	n.d.	n.d.	n.d.	ФN315, ФSa119, ФNM3, ФBU01 (*sea*)	n.d.	85.14% (*sed*, *selj ser*)

^1^ Reference sequences were obtained from GenBank. Accession number U93688.2; ^2^ CP000046.1, region 1,922,358–1,922,544; ^3^ CP000046.1, region 2,074,546–2,075,382; ^4^ CP000046.1, region 903,332–906,333; ^5^ CP000046.1, region 397,869–398,153; ^6^ AB983199.1; ^7^ AF217235.1; ^8^ NZ_LJBK01000031.1; ^9^ GQ900447.1; n.d.: not detected.

**Table 8:** Enterotoxins production in vivo: immunochemical qualitative and quantitative methods.

Batch and Strain	SEs Genes Detected	Milk after 18 h Incubation	Sampling Area	Qualitative Methods	Amount of Protein Produced [SE] (ng/g of Cheese) ^a^
1—ProNaCC1	*sed*/*ser*/*selj*	pos	Core	pos	[SED] = 7.966
			Periphery	pos	[SED] = 6.607
2—ProNaCC2	*see*	pos	Core	pos	[SEE] = 9.126
			Periphery	pos	[SEE] = 8.419
3—ProNaCC4	*sea*	pos	Core	pos	[SEA] = 2.760
			Periphery	pos	[SEA] = 2.648
4—ProNaCC5	*seg*/*sei*	neg	Core	neg	neg
			Periphery	neg	neg
5—ProNaCC6	*seh*	neg	Core	neg	neg
			Periphery	neg	neg
6—ProNaCC7	*sea*/*sed*/*selj*/*ser*	pos	Core	pos	[SEA] = 1.833
					[SED] = 7.578
		pos	Periphery	pos	[SEA] = 1.849
					[SED] = 7.841

^a^ Data are the means of two biological repetitions.

**Table 9:** Enterotoxins production in vivo: immunochemical qualitative and quantitative methods.

Batch and Strain	CPS in Spiked Milk 18 h Enrichment (CFU/g)	CPS in Fresh Cheese after Sweating (CFU/g)
1—ProNaCC1	2.1 × 10^7^	2.8 × 10^8^
2—ProNaCC2	5.3 × 10^7^	2.5 × 10^8^
3—ProNaCC4	1.1 × 10^8^	2.9 × 10^8^
4—ProNaCC5	9.2 × 10^7^	5.6 × 10^8^
5—ProNaCC6	9.2 × 10^7^	2.5 × 10^8^
6—ProNaCC7	3.8 × 10^7^	3.2 × 10^8^

## Supplementary Materials

The following are available online at https://www.mdpi.com/2073-4425/11/1/33/s1, Database S1: Multifasta file containing the sequences of the virulence factors genes included in this study; Table S2: List of the strains used for building the pan-genome of enterotoxigenic *S. aureus*. Database S3: Multifasta file containing the 99 sequences of staphylococcal enterotoxins genes and their variants included in the ABRicate database; Table S4: Prophage most common regions within the six enterotoxigenic genomes detected in this study; Database S5: Multifasta file containing the 74 sequences of SaPIs included in the ABRicate database; Table S6: Details of the SaPIs detected in the samples with an identity percentage higher than 95.0; Database S7: Multifasta file containing the sequences of *S. aureus* plasmids included in the ABRicate database; Table S8: Details of the plasmids detected; Table S9: Sequences detected of bacteriocins and locus tags; Table S10: List of all the virulence factors detected and locus tags. Table S11: MIC values for all the antimicrobial tested in this study.
